# Longitudinal Trends and Analytical Consistency of Folate and Vitamin B_12_ Biomarkers: Two Decades of Population-Based Data and Diagnostic Implications

**DOI:** 10.3390/biomedicines14010140

**Published:** 2026-01-09

**Authors:** Kristina Sejersen, Anders O. Larsson

**Affiliations:** 1 Department of Medical Sciences, Uppsala University, Uppsala University Hospital, 75185 Uppsala, Sweden; 2 Unilabs AB, 17154 Stockholm, Sweden

**Keywords:** vitamin B_12_, cobalamin, folate, vitamin B_9_, one-carbon metabolism, biomarkers, laboratory standardization, longitudinal analysis, clinical laboratory techniques

## Abstract

**Background/Objectives**: Vitamin B_12_ (cobalamin) and folate (vitamin B_9_) are essential cofactors in one-carbon metabolism required for DNA synthesis, methylation, and genomic stability. Deficiencies in these nutrients can cause megaloblastic anemia, neurological dysfunction, and hyperhomocysteinemia, linking micronutrient imbalance to cardiovascular and neurocognitive outcomes. Population-based surveillance of these biomarkers provides insight into nutritional trends and supports analytical standardization. **Methods**: This retrospective study included all routine plasma (P) vitamin B_12_ and folate measurements performed at Uppsala University Hospital from 2005 to 2024 (n = 647,302 and 578,509, respectively). Data were extracted from the laboratory information system and summarized using annual medians, percentile distributions, and coefficients of variation (CV). Linear regression was used to validate the method comparison and assess the impact of the 2021 transition from the Abbott Architect to the Roche cobas platform. Descriptive statistics summarized the temporal and seasonal patterns of P-vitamin B_12_ and P-folate. **Results**: Median P-vitamin B_12_ concentrations remained stable (340–370 pmol/L; median CV = 4.6%), while P-folate increased from 10.5 to 15.5 nmol/L (median CV = 12.9%) from 2005 to 2024. Low P-folate (<7 nmol/L) was observed in 7.1% of measurements and low or borderline P-vitamin B_12_ (<250 pmol/L) in 22.6%. Females exhibited slightly higher concentrations of both analytes. Although no clear seasonal pattern was observed, small biological effects cannot be excluded. Sample volumes decreased during the summer. The transition to Roche assays introduced measurable methodological shifts, particularly for P-folate. **Conclusions**: Levels of P-vitamin B_12_ remained stable over two decades, while P-folate status increased modestly. This reflects both dietary influences and assay-related differences following the 2021 platform transition. Continuous surveillance of biomarker medians provides a sensitive tool for detecting analytical drift and for monitoring long-term nutritional trends in clinical populations.

## 1. Introduction

Folate (vitamin B_9_) and vitamin B_12_ (cobalamin) deficiencies are significant global health challenges that frequently affect at-risk populations, such as children, pregnant women, and individuals facing socioeconomic disadvantages. There is considerable overlap between these deficiencies among these groups [1,2,3,4,5].

Vitamin B_12_ and folate are essential, water-soluble vitamins that play central roles in one-carbon metabolism. They support the transfer of methyl groups, which is necessary for DNA synthesis, methylation, and cellular repair [6,7,8]. These interrelated pathways are fundamental to hematopoietic function, neurodevelopment, and genomic stability. Disturbances in either vitamin disrupt homocysteine (Hcy) remethylation and S-adenosylmethionine-dependent methylation processes, linking micronutrient status to widespread biochemical and molecular effects [6,7].

Deficiencies in vitamins B_12_ and folate can lead to megaloblastic anemia, neurocognitive dysfunction, and elevated Hcy levels, which are an established risk factor for cardiovascular and neurodegenerative diseases [9,10,11]. A vitamin B_12_ deficiency is associated with demyelination, axonal degeneration, and impaired neurotransmitter synthesis. Folate insufficiency, on the other hand, contributes to defective nucleotide metabolism and chromosomal instability [9,10]. Recent advances in molecular biology have deepened our understanding of these mechanisms, highlighting the connection between methylation capacity, DNA integrity, and neurological health [6].

Plasma (P) concentrations of vitamin B_12_ and folate are commonly used as indicators of nutritional status in clinical and epidemiological settings. However, the interpretation of these biomarkers is complicated by inter-assay variability, differences in reagent formulation, and a lack of full analytical standardization across platforms [12,13,14]. Although total P-vitamin B_12_ is the most common indicator in routine practice, functional biomarkers such as holotranscobalamin (holo-TC), methylmalonic acid (MMA), and Hcy offer additional insights into cellular metabolism [12]. Similarly, P-folate reflects recent intake, whereas red blood cell folate more accurately represents long-term stores [13]. On-going efforts toward assay harmonization and the development of standardized reference materials aim to improve comparability between laboratories over time [14]. Standard quality control activities are effective at identifying acute errors but often fail to reveal gradual changes or persistent trends. Tracking median patient values in addition to these methods offers an additional tool for recognizing analytical problems under routine conditions [15]. All analyses in the present study were performed at the measurement level, and individual patients could contribute more than one test result over time.

Ongoing surveillance of median population levels of P-vitamin B_12_ and P-folate offers valuable insight into large-scale metabolic patterns and the sustained reliability of laboratory analytics. Monitoring long-term trends in these biomarkers helps us assess nutritional status, dietary changes, and the impact of public health initiatives. This approach is especially important in Northern Europe, where mandatory folic acid fortification has not been adopted, so regular monitoring is crucial for understanding population health [11,16]. Contemporary studies have highlighted the association between P-vitamin B_12_ and P-folate levels and chronic disease outcomes, including metabolic dysfunction and all-cause mortality. These studies emphasize the broader prognostic importance of these nutrients [17,18].

Several large, laboratory- and population-based studies have reported on vitamin B_12_ and folate distributions, deficiency prevalence, and shorter-term temporal patterns in different regions [1,19,20]. However, most of these studies have been cross-sectional or have been limited to specific subgroups, survey cohorts, or relatively short follow-up periods [16,21]. In contrast, the present study uses nearly two decades of routine P-vitamin B_12_ and P-folate measurements from a single tertiary-care laboratory to examine long-term temporal trends and seasonal patterns, assess the influence of analytical platform transitions, and evaluate the utility of long-term median- and percentile-based monitoring for nutritional surveillance and the detection of analytical drift in a real-world clinical setting.

## 2. Materials and Methods

### 2.1. Samples

Routine clinical requests for P-vitamin B_12_ and P-folate at the Department of Clinical Chemistry, Uppsala University Hospital, were collected in lithium heparin plasma tubes (BD Vacutainer, ref. 367962; BD Vacutainer Systems, Plymouth, UK) for analysis. Data were retrieved from the laboratory information system without full patient identifiers, in accordance with ethical approval. The dataset included sampling month and year, patient age (in years), sex, and P-vitamin B_12_ and P-folate concentrations. Each record in the dataset represents a single laboratory measurement. All eligible routine test results were included, regardless of whether the measurement was repeated for an individual.

#### 2.1.1. Data Cleaning and Exclusions

The dataset was exported from the laboratory information system after routine instrument and middleware validation was completed, which included auto-verification rules for P-vitamin B_12_ and P-folate including that internal controls were within defined intervals. Measurements with technical error flags or values outside the analytical reportable range were automatically prevented from being released and were therefore not included in the export. In accordance with ethical approval, only age and sex were available as patient-level variables. After extraction, the only additional exclusion was the removal of a small number of manually entered results with incorrect numeric values. All remaining records contained valid age and sex information, fell within the laboratory’s validated measuring interval, and passed internal quality control. Therefore, no further filtering was applied.

#### 2.1.2. Definition of P-Vitamin B_12_ and P-Folate Deficiency and Insufficiency

Concentrations were interpreted according to established clinical thresholds, where P-folate < 7.0 nmol/L indicates folate deficiency and P-vitamin B_12_ < 100 pmol/L indicates vitamin B_12_ deficiency. P-vitamin B_12_ < 250 pmol/L was considered low or borderline, reflecting biochemical insufficiency. These decision limits are consistent with international and regional clinical guidelines and key reviews, which indicate that overt vitamin B_12_ deficiency is typically observed below approximately 100–150 pmol/L, whereas metabolic abnormalities in MMA and Hcy become increasingly common below approximately 250–275 pmol/L [12,22,23]. For folate, a plasma concentration below 7 nmol/L is widely used in European and international guidance as the threshold associated with an increased risk of megaloblastic anemia [11,16,24].

### 2.2. Laboratory Methods

Throughout the study period, the concentrations of P-vitamin B_12_ and P-folate were analyzed using standardized laboratory protocols. Initially, all assays were performed on an Architect i2000 analyzer (Abbott Laboratories, Abbott Park, IL, USA). P-vitamin B_12_ was analyzed with reagent 6C09 until the end of 2018, when reagent 7K61-25 (Abbott Laboratories) was introduced. P-folate was measured using reagent 7K60-32 until the end of 2010, then reagent 1P74-35 (Abbott Laboratories) was applied.

In 2021, P-vitamin B_12_ and P-folate analyses were transferred to a cobas e 801 analyzer (Roche Diagnostics, Rotkreuz, Switzerland). P-vitamin B_12_ was analyzed using the cobas Elecsys Vitamin B_12_ II reagent kit (ref. 07028121190, Roche Diagnostics), and P-folate using the cobas Elecsys Folate III reagent kit (ref. 07027290190, Roche Diagnostics). Method verification was performed during the transition from the Abbott to the Roche analytical systems.

For P-vitamin B_12_, mean results showed no overall bias (0% difference), although individual samples deviated by more than ±10%. In external quality assessment (Equal-is/Labquality), the Roche group averaged 9% higher than our results during the past year. Coefficients of variation (CV%) for internal controls during implementation were within accredited performance criteria.

In Equalis/Labquality external quality assessment, the Roche Folate III group averaged 27% lower than our results on lyophilized serum materials over the past year. On fresh patient samples, P-folate concentrations were on average 6% higher on cobas platform, although individual deviations were larger. Internal control CVs during implementation remained within accreditation limits. The method was approved for routine use on cobas platform, maintaining the existing reference interval, consistent with Roche’s lower reference limit.

#### Method Comparison Between Platforms

Prior to the 2021 transition from the Abbott Architect i2000 (Abbott Laboratories, Abbott Park, IL, USA) to the Roche cobas pro e 801 (Roche Diagnostics, Rotkreuz, Switzerland), formal method comparisons were performed for P-vitamin B_12_ and P-folate using parallel patient samples as part of routine validation. Simple linear regression was used to assess agreement. For P-vitamin B_12_, the regression analysis yielded a slope close to 1.0, a small intercept, and a correlation coefficient greater than 0.98. These results indicate excellent agreement and no clinically significant systematic bias. Similarly, for P-folate, the regression analysis showed a high correlation (r > 0.97), with a slope close to 1.0 and a small intercept. This is consistent with a modest proportional and/or constant difference that remained within the predefined acceptance limits for clinical use.

Since the validation demonstrated acceptable comparability, no post hoc recalibration or regression-based harmonization of historical results was applied. Instead, figures indicate the timing of the analytical platform transition in 2021 and consider potential assay-related contributions to apparent P-folate shifts from 2021 to 2024 when interpreting long-term trends.

### 2.3. Statistical Analyses

Statistical analyses, including linear regression, were performed using Excel 365 (Microsoft Corporation, Seattle, WA, USA) and Statistica version 10 (Tibco Software, Palo Alto, CA, USA). Simple linear regression was used exclusively for method comparison validation between the Abbott Architect and Roche cobas platforms, as well as for descriptive characterization of temporal trends. Data are presented as median, lower quartile, upper quartile, 10th percentile, and 90th percentile. Since P-vitamin B_12_ and P-folate concentrations in large laboratory populations are usually right-skewed and affected by outliers [11], we report medians with interquartile ranges. These provide a more reliable and representative measure of central tendency than means do for skewed data. Categorical variables are expressed as counts and percentages of the total. Additionally, Spearman’s rank-order correlation was used to evaluate monotonic temporal trends by correlating calendar year with the annual median and selected percentile values (10th, 25th, 50th, 75th, and 90th) of P-vitamin B_12_ and P-folate.

### 2.4. Ethics

The study was approved by the Regional Ethical Review Board in Uppsala, Sweden (Dnr 01-367) in accordance with national regulations governing research involving human data and biological samples. Because the study was a retrospective analysis of previously collected, fully de-identified laboratory data with only age and sex available as patient-level variables, the Ethics Committee granted a waiver of individual informed consent.

## 3. Results

### 3.1. Baseline Characteristics

From 1 January 2005, to 31 December 2024, a total of 647,302 P-vitamin B_12_ measurements and 578,509 P-folate measurements were reported.

Among males, 228,226 (35%) P-vitamin B_12_ measurements and 202,571 (35%) P-folate measurements were recorded. Among females, 419,076 (65%) P-vitamin B_12_ and 375,938 (65%) P-folate measurements were recorded. The median age of males contributing P-vitamin B_12_ measurements was 64 years (interquartile range (IQR): 42–76), while the median age of females was 53 years (IQR: 34–73). For P-folate measurements, the median age was 63 years (IQR: 40–76) for males and 52 years (IQR: 33–73) for females (Table 1).

The median P-vitamin B_12_ concentration was 340 pmol/L (interquartile range (IQR): 254–484) in males and 349 pmol/L (IQR: 260–499) in females, indicating slightly higher levels in females. The median P-folate concentration was 13.8 nmol/L (IQR: 9.7–22.7) in males and 15.2 nmol/L (IQR: 10.5–25.7) in females; again, the levels were higher in females (Table 2). These findings demonstrate modest but consistent sex-related differences in P-vitamin B_12_ and P-folate concentrations.

Overall, 41,111 P-folate measurements out of 578,511 (7.1%) were below 7.00 nmol/L, indicating low P-folate levels. For P-vitamin B_12_, 2587 measurements out of 647,302 (0.4%) were below 100 pmol/L and 146,373 measurements (22.6%) were below 250 pmol/L (including those below 100 pmol/L), which reflects biochemical evidence of deficiency or borderline status. Concentrations were interpreted according to established clinical thresholds, where P-folate < 7.0 nmol/L and P-vitamin B_12_ < 100 pmol/L indicate deficiency. P-vitamin B_12_ < 250 pmol/L reflects low or borderline status [11,16,17].

Median P-vitamin B_12_ and P-folate concentrations were slightly higher in females than in males: median P-vitamin B_12_ was 349 versus 340 pmol/L and median P-folate was 15.2 versus 13.8 nmol/L (Table 2), reflecting modest sex-related differences in this large cohort.

### 3.2. Changes in the Number of Requests for P-Vitamin B_12_ and P-Folate over Time

**Figure 1:**
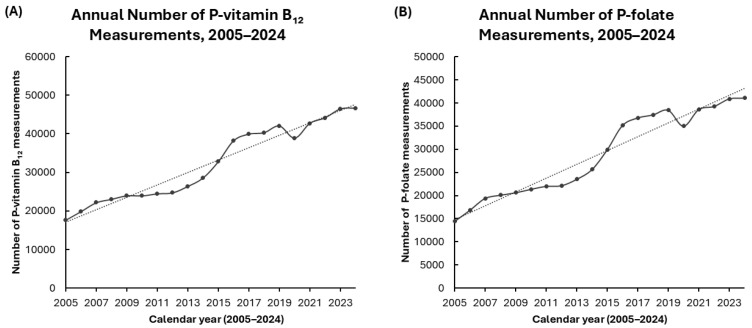
Annual numbers of P-vitamin B_12_ and P-folate measurements from 2005 to 2024. Legend: (**A**) Number of P-vitamin B_12_ measurements per year. (**B**) Number of P-folate measurements per year. Each point represents the total number of routine plasma measurements reported by the laboratory in a given calendar year. Dotted lines indicate linear trends over time.

From 2005 to 2024, the annual number of P-vitamin B_12_ measurements increased substantially, with a marked rise observed after 2015. This rise coincided with the implementation of updated regional and national guidelines for detecting and managing vitamin B_12_ deficiency [22,25,26,27,28,29]. A similar trend was observed for P-folate, with measurement volumes demonstrating a continuous upward trajectory and reaching their peak in recent years. The measurement volumes for both P-vitamin B_12_ and P-folate decreased in 2020, coinciding with the onset of the COVID-19 pandemic [30]. This decline aligns with the disruption of routine healthcare services and reduced laboratory utilization documented during the early phase of the pandemic. Subsequently, measurement volumes rebounded rapidly, resuming the prior upward trend (Figure 1).

These findings suggest a temporal association between increased testing and the introduction of new clinical recommendations, including broader diagnostic criteria and the adoption of molecular diagnostic approaches. The 2020 decrease reflects the pandemic’s impact on routine laboratory activities, as reported in multiple studies. While this dataset did not establish a direct statistical correlation, the parallel rise in testing volumes following guideline updates highlights the likely influence of evolving clinical practice on laboratory utilization.

### 3.3. Changes in P-Vitamin B_12_ and P-Folate Concentrations over Time

#### 3.3.1. Longitudinal Trends in P-Vitamin B_12_ and P-Folate (2005–2024)

**Figure 2:**
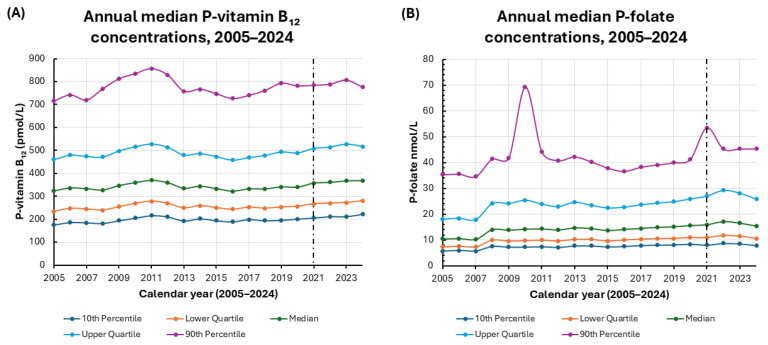
Temporal distributions of P-vitamin B_12_ and P-folate concentrations from 2005 to 2024. Legend: (**A**) P-vitamin B_12_ concentrations by year, showing the median, interquartile ranges (lower and upper quartiles), and the 10th and 90th percentiles. (**B**) P-folate concentrations by year, displaying the median, lower and upper quartiles, and the 10th and 90th percentiles All annual distributions are based on the total number of plasma measurements performed that year. Note that individual patients may contribute more than one measurement. The vertical line at 2021 shows the switch from the Abbott Architect i2000 to the Roche cobas pro e 801.

A longitudinal analysis of P-vitamin B_12_ concentrations from 2005 to 2024 revealed overall temporal stability with minor fluctuations over the two-decade period (Figure 2A). The median P-vitamin B_12_ level remained relatively constant, ranging from 322 pmol/L in 2016 to 370 pmol/L in 2011 and reaching 369 pmol/L in 2024. IQR remained narrow throughout the observation period. Lower quartile values ranged from 234 pmol/L in 2005 to 280 pmol/L in 2024. Upper quartile values ranged from 458 pmol/L in 2016 to 527 pmol/L in 2011 and 2023. This indicates a consistent central distribution over time.

At the lower end of the distribution, the 10th percentile increased gradually from 175 pmol/L in 2005 to 222 pmol/L in 2024, suggesting a stable prevalence of low P-vitamin B_12_ concentrations. In contrast, the 90th percentile showed greater temporal variation, reaching peak values of 856 pmol/L in 2011 and secondary elevations of 781–806 pmol/L from 2019 to 2023. These transient increases declined to 776 pmol/L in 2024, possibly reflecting short-term, population-level changes in supplementation, dietary fortification, or analytical variability. Overall, P-vitamin B_12_ concentrations have remained stable over the past two decades. There have been only modest, transient increases at the upper end of the distribution. These small absolute changes are unlikely to be of major clinical relevance at the population level.

In parallel, a longitudinal analysis of P-folate concentrations from 2005 to 2024 revealed a gradual upward trend with modest fluctuations across the percentile distribution (Figure 2B). The median P-folate increased from 10.5 nmol/L in 2005 to 15.5 nmol/L in 2024, indicating a moderate elevation in overall P-folate levels. The lower and upper quartiles also increased over time, rising from 7.4 and 18.1 nmol/L in 2005 to 10.7 and 25.9 nmol/L in 2024, respectively. This suggests a rightward shift in the entire distribution.

The 10th increased progressively from 5.7 nmol/L in 2005 to 8.0 nmol/L in 2024, reflecting a mild rise in baseline concentrations among the lowest values. The 90th percentile exhibited intermittent peaks, notably in 2010 (69.4 nmol/L) and from 2021 to 2023 (45.4–53.6 nmol/L), followed by a return to moderate levels in 2024 (45.4 nmol/L). These transient high-end elevations likely reflect episodic influences, such as shifts in population demographics, sampling patterns, or dietary supplementation, rather than sustained population-level changes. Overall, P-folate concentrations exhibited modest long-term increases with stable distributional structures and periodic variability at the upper percentiles. Although the absolute changes were statistically detectable, they were small and should be interpreted as having limited clinical impact on individual patients. Table 3 summarizes the correlations between the calendar year and the annual percentiles of P-vitamin B_12_ and P-folate.

#### 3.3.2. Coefficient of Variation Analysis for P-Vitamin B_12_ and P-Folate

To evaluate temporal consistency, we calculated the coefficients of variation (CV%) for P-vitamin B_12_ and P-folate across selected summary statistics (Table 4). P-vitamin B_12_ exhibited low CV values across all metrics, indicating minimal interannual variability and high analytical reproducibility. The CV for the median was 4.61%, with similar values across quartiles (4.48–5.10%) and slightly higher dispersion at the 10th percentile (6.16%), which confirms overall temporal stability.

In contrast, P-folate demonstrated substantially higher interannual variability, with CV values approximately two- to three-fold greater than those of P-vitamin B_12_. The median CV for P-folate was 12.94%, and the greatest variability was observed at the 90th percentile (18.18%), suggesting that circulating P-folate levels are more sensitive to external influences, including dietary intake, fortification policies, and analytical differences.

Together, these results suggest that P-vitamin B_12_ concentrations have remained stable over the past two decades while P-folate levels have moderately increased and displayed higher temporal variability. The low CV values for P-vitamin B_12_ support the reliability and reproducibility of long-term, population-level monitoring of this biomarker.

### 3.4. Seasonal Variation of P-Vitamin B_12_ and P-Folate Concentrations

**Figure 3:**
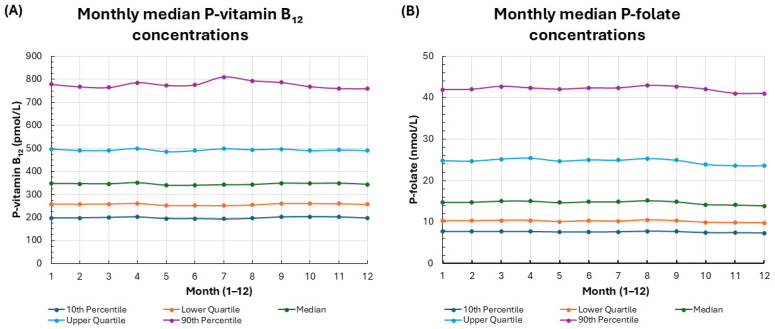
Monthly distributions of P-vitamin B_12_ and P-folate concentrations. Legend: (**A**) P-vitamin B_12_ concentrations by month. (**B**) P-folate concentrations by month. Median values, interquartile ranges (lower and upper quartiles), and the 10th–90th percentiles are shown.

Upon visual inspection, the data showed no clear seasonal pattern for P-vitamin B_12_ and P-folate analytes. The monthly medians, interquartile ranges, and 10th–90th percentiles for these analytes appeared consistent throughout the year (Figure 3A,B), with subtle month-to-month fluctuations and no obvious systematic peaks or troughs. This suggests minor seasonal effects that cannot be fully excluded.

**Figure 4:**
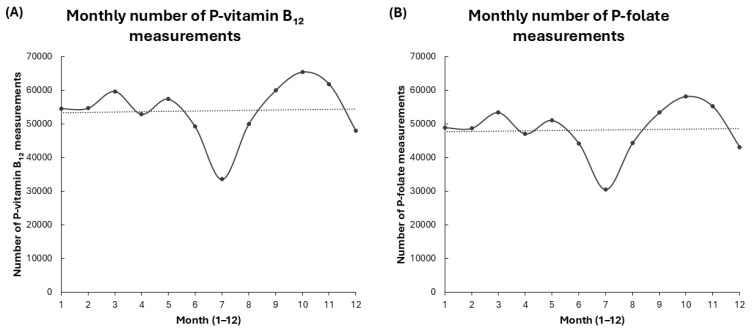
Monthly distribution of P-vitamin B_12_ and P-folate measurements. Legend: (**A**) Number of P-vitamin B_12_ measurements per month. (**B**) Number of P-folate measurements per month. The dashed horizontal line shows the average number of monthly measurements taken throughout the entire study period.

However, the number of test requests for both P-vitamin B_12_ and P-folate demonstrated a consistent seasonal pattern: a pronounced decline during the summer months (June–August), with a minimum in July, followed by an increase in the spring and autumn (Figure 4A,B). Despite the seasonal variation in testing frequency, the underlying concentrations of biomarkers remained largely stable, suggesting that levels of P-vitamin B_12_ and P-folate are minimally affected by seasonal factors.

Together, these findings suggest that P-vitamin B_12_ and P-folate levels remain stable in the population throughout the year, despite pronounced seasonal variation in testing.

## 4. Discussion

In this large-scale, two-decade longitudinal study of routine clinical data, we observed stability in P-vitamin B_12_ concentrations and modest long-term increases in P-folate concentrations among individuals whose routine samples were analyzed at a tertiary care laboratory in Uppsala, Sweden. Spearman’s rank-order correlation revealed an absence of consistent, monotonic trends in annual P-vitamin B_12_ percentiles. In contrast, P-folate exhibited strong positive correlations with the calendar year for all evaluated percentiles. These results suggest a gradual, widespread increase in P-folate levels throughout the study period, rather than isolated changes in specific parts of the distribution. This pattern is consistent with the combined effects of analytical and population-level factors on P-folate concentrations over time. Overall, these findings imply that P-vitamin B_12_ levels have remained relatively constant at the population level while P-folate levels have gradually increased. However, the magnitude of these changes is modest, so the statistical significance of the trends does not necessarily translate into substantial clinical effects for individual patients. This likely reflects changes in dietary habits, supplementation practices, or analytical methods over time [11,22,24]. The transition from the Abbott Architect platform to the Roche cobas pro e 801, as well as the introduction of the Roche Elecsys Folate III assay, may have contributed to assay-dependent shifts in measured P-folate concentrations. Therefore, changes observed since 2021 should be interpreted with this platform transition in mind.

Beyond these assay-dependent shifts, the gradual increase in P-folate concentrations from 2005 to 2023 suggests an improvement in population P-folate status over the past two decades. The modest decline in 2024 coincided with the transition to the Roche Diagnostics Folate III assay, which has been reported to yield lower results than previous methods [31]. Therefore, this particular apparent decrease in P-folate concentrations is likely due to methodology rather than biology. Taken together, the longitudinal data indicate a sustained improvement in P-folate status. However, the observed increases in P-folate concentrations are small, so the distinction between statistical and clinical significance should be kept in mind when interpreting these temporal trends. Recent assay-dependent shifts highlight the importance of methodological harmonization when interpreting temporal trends.

Measuring P-folate concentration remains analytically challenging because it encompasses several interrelated folate compounds rather than a single molecule. While circulating folate is primarily 5-methyltetrahydrofolate, other forms, such as 5-formyl- and 10-formyltetrahydrofolate, also contribute to total concentrations [17,32]. The sensitivity and specificity of assay methods differ for these forms, which hinders comparability and standardization across laboratories [16,17,19,32]. International efforts led by the World Health Organization (WHO), Centers for Disease Control and Prevention (CDC), and National Institute of Standards and Technology (NIST) aim to harmonize folate measurements through liquid chromatography–mass spectrometry (LC–MS/MS)-based reference procedures. However, full standardization has yet to be achieved [16,19].

Disturbances in either vitamin can disrupt Hcy remethylation and S-adenosylmethionine-dependent methylation. This links micronutrient status to genomic stability, epigenetic regulation, and cellular proliferation. Therefore, monitoring these biomarkers provides insights into both nutritional and molecular aspects of metabolic health.

The absence of significant change in median P-vitamin B_12_ plasma concentrations from 2005 to 2024–with annual medians ranging only from 322 to 370 pmol/L and a coefficient of variation of 4.6–indicates substantial biological and analytical stability. This finding is consistent with those from other Northern European regions without folic acid fortification [20,21]. In contrast, gradual increases in P-folate concentrations across percentiles suggest improved folate availability. Recent reviews point to the increased use of fortified foods and supplements, as well as enhanced assay sensitivity, as potential contributors to these upward shifts [7]. Folate exhibited greater temporal variability than vitamin B_12_, supporting the idea that circulating folate levels are more sensitive to dietary and methodological influences [12].

Although population-level P-vitamin B_12_ concentrations have remained stable, deficiency remains clinically significant due to its role in maintaining neurological function. Molecular studies have revealed how cobalamin deficiency impairs myelin formation, induces axonal degeneration, and alters neurotransmitter synthesis via methylation-dependent pathways [6,9]. At the cellular level, folate deficiency has been shown to trigger genomic instability and aberrant DNA replication by impairing thymidylate synthesis and causing uracil to be misincorporated into DNA [33]. Conversely, higher P-folate concentrations, as observed here, may promote enhanced genomic maintenance and methylation balance at the population level.

The prevalence of low P-folate (7.1%) and suboptimal P-vitamin B_12_ levels (22.6%) in this study is consistent with patterns observed in other large-scale biochemical surveys of European populations. In absolute terms, 41,111 out of 578,509 P-folate measurements (7.1%) were below 7.0 nmol/L, suggesting that severe folate deficiency is uncommon. Meanwhile, 146,373 out of 647,302 P-vitamin B_12_ measurements (22.6%) were in the low or borderline range. These figures reflect the clinical testing population rather than the general population [1,19]. These prevalence estimates are specific to the decision limits applied in this study. Using higher thresholds, such as ≥300 pmol/L for vitamin B_12_ or ≥10 nmol/L for folate, as recommended in certain guidelines and expert reviews [16,23,34], would result in a higher percentage of measurements being classified as low or insufficient. Conversely, lower thresholds would reduce prevalence, though they may also underestimate early or subclinical deficiency [12,23]. While overt deficiency is uncommon, marginal status remains a significant public health concern [11,17,35]. The low proportion of measurements below 7.0 nmol/L indicates that folate deficiency is relatively rare, likely due to improved dietary folate intake and supplementation practices in recent years.

In contrast, vitamin B_12_ insufficiency was more prevalent: 0.4% of samples had concentrations below 100 pmol/L, and 22.6% had concentrations below 250 pmol/L, encompassing both deficient and borderline states. These thresholds correspond to established cut-offs for biochemical deficiency, which can occur before the onset of hematological abnormalities, yet still confer risk for neurological or cognitive dysfunction [23,36,37]. Since even mild insufficiency can contribute to neurocognitive impairment and elevated Hcy concentrations, these results underscore the importance of assessing vitamin B_12_ status beyond traditional anemia screening for clinical and preventive purposes.

Because total P-vitamin B_12_ alone often fails to detect early metabolic deficiencies, the use of functional markers is becoming more widely accepted. Holo-TC, which represents the biologically active fraction of circulating B_12_, decreases before total B_12_ concentrations decline. This improves diagnostic sensitivity [34]. Elevated levels of MMA and Hcy further indicate impaired intracellular metabolism. MMA is relatively specific to vitamin B_12_ deficiency, while Hcy responds to both folate and vitamin B_12_ insufficiency [38]. Integrating these markers, such as through the combined B_12_ status index (cB_12_), offers greater discriminatory power for detecting functional deficiency [39].

The relatively low prevalence of folate deficiency compared to vitamin B_12_ insufficiency reflects trends observed in northern European populations. This is likely due to improved folate nutrition resulting from supplementation and fortified foods [21]. However, the widespread borderline vitamin B_12_ status observed underscores the need for improved diagnostic strategies and early intervention, particularly among at-risk groups, including older adults, vegetarians, and individuals with gastrointestinal disorders or malabsorption.

Overall, the current evidence suggests that, although folic acid fortification is highly effective in reducing neural tube defects, high supplemental intake above physiological needs may mask or exacerbate vitamin B_12_-deficient neuropathy. To balance nutritional adequacy with safety, continued monitoring of folate status alongside active vitamin B_12_ markers (holo-TC and MMA) is recommended [40,41,42,43,44].

Consistent with previous observations in population-based cohorts, both P-vitamin B_12_ and P-folate concentrations were modestly higher in females than in males [11,35,45]. However, the absolute differences were small and unlikely to be of major clinical relevance at the individual level, despite being detectable in this large dataset. These differences may reflect physiological and behavioral factors, including higher supplement use and dietary pattern differences among women [46]. The absolute median differences were small: 9 pmol/L for P-vitamin B_12_ (349 vs. 340 pmol/L) and 1.4 nmol/L for P-folate (15.2 vs. 13.8 nmol/L). Although these differences are statistically detectable, they are unlikely to influence diagnostic classification at the individual level [12,23]. However, the small absolute differences observed suggest limited clinical significance at the population level.

During the study period, analytical transitions from Abbott Architect to Roche cobas systems introduced potential systematic shifts, particularly for P-folate, as indicated by external quality assessments. Despite these differences, internal quality remained within accredited limits, which supports the validity of the observed trends. Interpreting P-vitamin B_12_ biomarkers is complex because total P-vitamin B_12_ does not always reflect functional status. Complementary markers, such as holo-TC, MMA, and Hcy, provide greater specificity [12]. Nevertheless, total P-vitamin B_12_ remains the most practical and standardized parameter for long-term surveillance.

The significant rise in P-vitamin B_12_ and P-folate testing after 2015 likely reflects updated national and regional guidelines that promote earlier detection of vitamin B_12_ deficiency. The decline in 2020 was temporary and corresponded with the disruption of routine healthcare utilization worldwide due to the pandemic. Beyond their diagnostic role, circulating folate and vitamin B_12_ levels have been associated with metabolic and cardiovascular outcomes, emphasizing their relevance as prognostic and public health biomarkers [47]. The stability of these analytes thus indicates an overall consistent nutritional status in the Swedish population over the past two decades.

Upon visual inspection, the monthly median concentrations of P-vitamin B_12_ and P-folate did not exhibit a clear, systematic seasonal pattern, which is consistent with biological stability across seasons. However, small seasonal effects cannot be ruled out. These results are consistent with previous studies indicating that these vitamins are minimally impacted by seasonal dietary or environmental factors in populations living in high latitudes [8,48].

### Strengths, Limitations, and Implications

The major strengths of this study include the exceptionally large dataset of nearly one million P-vitamin B_12_ and P-folate measurements collected over two decades and consistent laboratory quality assurance throughout the study period. These features enabled a precise assessment of long-term and seasonal trends in biomarker distributions. Limitations include the retrospective design, the absence of dietary and supplement information, and the potential effects of analytical platform transitions. Additionally, since the data were derived from clinical test requests rather than population screening, the sample may overrepresent individuals with suspected deficiency, which limits the study’s generalizability to the general population. Furthermore, since analyses were performed at the measurement level, individuals who were tested frequently may be overrepresented compared to the general population. This could introduce minor bias into the aggregated results.

## 5. Conclusions

Overall, this longitudinal analysis demonstrates the long-term stability of P-vitamin B_12_ concentrations, as well as a modest increase in folate status, within a large Swedish clinical population with minimal seasonal variation. The findings suggest stable micronutrient status despite significant changes in healthcare utilization and analytical methodology. These results underscore the value of laboratory archives for nutritional surveillance and highlight the importance of ongoing assay harmonization to accurately monitor long-term biomarker trends.

## Figures and Tables

**Table 1:** Age distributions by sex and analysis type.

Analysis	Sex	Valid N (%)	Mean Age (Years)	Median Age (Years)	Lower Quartile (Years)	Upper Quartile (Years)	10th Percentile	90th Percentile
P-vitamin B_12_	All	647,302 (100%)	56	59	36	75	23	83
	Men	228,226 (35%)	59	64	42	76	24	84
	Women	419,076 (65%)	53	53	34	73	23	83
P-folate	All	578,509 (100%)	55	56	35	74	22	84
	Men	202,571 (35%)	58	63	40	76	23	84
	Women	375,938 (65%)	53	52	33	73	22	83

Legend: Data are presented as number of measurements (valid N, %), mean and median age (years), interquartile range (lower and upper quartiles), and 10th and 90th percentiles.

**Table 2:** P-vitamin B_12_ and P-folate concentrations by sex.

Analysis (Units)	Group	Valid N (%)	Mean	Median	Lower Quartile	Upper Quartile	10th Percentile	90th Percentile
P-vitamin B_12_ (pmol/L)	All	647,302 (100%)	433	348	258	496	200	770
	Men	228,226 (35%)	422	340	254	484	198	742
	Women	419,076 (65%)	439	349	260	499	201	797
P-folate (nmol/L)	All	578,509 (100%)	21.0	14.7	10.2	24.7	7.6	42.1
	Men	202,571 (35%)	20.3	13.8	9.7	22.7	7.3	41.9
	Women	375,938 (65%)	21.4	15.2	10.5	25.7	7.8	42.2

Legend: Data are presented as number of measurements (valid N, %), mean and median concentrations, interquartile range (lower and upper quartiles), and 10th and 90th percentiles. P-vitamin B_12_ concentrations are reported in pmol/L and P-folate in nmol/L.

**Table 3:** Spearman rank-order correlations between calendar year and annual percentiles of P-vitamin B_12_ and P-folate concentrations.

Analysis	Percentile	n	Spearman’s R	T (N-2)	*p*-Value
P-Vitamin B_12_	0.10	20	0.659	3.72	0.002
	0.25	20	0.568	2.93	0.009
	0.50	20	0.386	1.78	0.093
	0.75	20	0.394	1.82	0.086
	0.90	20	0.253	1.11	0.283
P-folate	0.10	20	0.891	8.34	<0.001
	0.25	20	0.916	9.68	<0.001
	0.50	20	0.893	8.43	<0.001
	0.75	20	0.742	4.70	<0.001
	0.90	20	0.462	2.21	0.040

Spearman’s rank-order correlation was used to calculate correlations, which were considered statistically significant at *p* < 0.05.

**Table 4:** Coefficient of variation (CV%) for P-vitamin B_12_ and P-folate according to selected summary statistics.

Summary Statistic	P-Vitamin B_12_ (CV%)	P-Folate (CV%)
10th percentile	6.16	11.47
Lower quartile	5.10	12.10
Median	4.61	12.94
Upper quartile	4.48	12.60
90th percentile	5.00	18.18

Abbreviation: CV, coefficient of variation.

## Data Availability

The corresponding author will make the data available upon reasonable request.

## Abbreviations

The following abbreviations are used in this manuscript:

P	Plasma
CV	Coefficient of Variation
IQR	Interquartile Range
holo-TC	Holotranscobalamin
MMA	Methylmalonic Acid
Hcy	Homocysteine
cB_12_	Combined B_12_ Status Index
LC–MS/MS	Liquid Chromatography–Tandem Mass Spectrometry
WHO	World Health Organization
CDC	Centers for Disease Control and Prevention
NIST	National Institute of Standards and Technology
COVID-19	Coronavirus Disease 2019
Equalis/LabQuality	External Quality Assessment Programs
